# Frequency of human immunodeficiency virus type-2 in hiv infected patients in Maputo City, Mozambique

**DOI:** 10.1186/1743-422X-8-408

**Published:** 2011-08-17

**Authors:** Cremildo Maueia, Deise Costa, Bindiya Meggi, Nalia Ismael, Carla Walle, Raphael Curvo, Celina Abreu, Nilesh Bhatt, Amilcar Tanuri, Ilesh V Jani, Orlando C Ferreira

**Affiliations:** 1 Instituto Nacional de Saúde, Maputo, Moçambique; 2 Laboratório de Virologia Molecular e Animal, Universidade Federal do Rio de Janeiro, Rio de Janeiro, Brazil; 3 Centro de Saúde do Alto-Maé, Maputo, Moçambique; 4 Laboratório de Histocompatibilidade e Criopreservação, Universidade do Estado do Rio de Janeiro, Rio de Janeiro, Brazil

**Keywords:** HIV-2, laboratory diagnosis, sub-Saharan Africa, Mozambique

## Abstract

The HIV/AIDS pandemic is primarily caused by HIV-1. Another virus type, HIV-2, is found mainly in West African countries. We hypothesized that population migration and mobility in Africa may have facilitated the introduction and spreading of HIV-2 in Mozambique. The presence of HIV-2 has important implications for diagnosis and choice of treatment of HIV infection. Hence, the aim of this study was to estimate the prevalence of HIV-2 infection and its genotype in Maputo, Mozambique.

HIV-infected individuals (N = 1,200) were consecutively enrolled and screened for IgG antibodies against HIV-1 gp41 and HIV-2 gp36 using peptide-based enzyme immunoassays (pepEIA). Specimens showing reactivity on the HIV-2 pepEIA were further tested using the INNO-LIA immunoblot assay and HIV-2 PCR targeting RT and PR genes. Subtype analysis of HIV-2 was based on the protease gene.

After screening with HIV-2 pepEIA 1,168 were non-reactive and 32 were reactive to HIV-2 gp36 peptide. Of this total, 30 specimens were simultaneously reactive to gp41 and gp36 pepEIA while two samples reacted solely to gp36 peptide. Only three specimens containing antibodies against gp36 and gp105 on the INNO-LIA immunoblot assay were found to be positive by PCR to HIV-2 subtype A.

The proportion of HIV-2 in Maputo City was 0.25% (90%CI 0.01-0.49). The HIV epidemic in Southern Mozambique is driven by HIV-1, with HIV-2 also circulating at a marginal rate. Surveillance program need to improve HIV-2 diagnosis and consider periodical survey aiming to monitor HIV-2 prevalence in the country.

## Introduction

Human immunodeficiency virus (HIV) type 2 (HIV-2) is a related but distinct virus from HIV-1, in spite of having similar gene products, genetic organization and cell tropism *in vitro*. HIV-2 also shows decreased cell killing, syncytium formation, and replication rates when compared with HIV-1. These features may justify the lower transmissibility of HIV-2, as well as its reduced pathogenicity and longer clinical course. Nevertheless, HIV-2 can cause immunosuppression and progression to AIDS [1-3]. HIV-2 was first isolated in West Africa in 1986 and has been reported in many countries of Western Africa such as the Gambia, Guinea, Ghana, Cape Verde, Guinea-Bissau, Cote d'Ivoire, Liberia, Senegal, and Niger [4,5]. Outside Africa, HIV-2 infection is sporadically detected in countries with socioeconomic relations with West Africa such as Portugal, Spain, France, United Kingdom and India [6-9]. The presence of HIV-2 is not regularly surveyed and generally assumed to be low in other African countries outside West Africa. However, in the last three decades there has been an increase in population migration within Africa, mainly attributed to regional wars, political upraises and even to increased international travel inside Africa [3,10].

The laboratory diagnosis of HIV-2 infection is challenged by the high cross-reactivity rate between the two viruses. Overall, HIV diagnosis is performed by a combination of one screening assay, often an Enzyme Immunoassay (EIA), followed by a confirmatory Western blot assay. However, the use of HIV-1 Western blots may lead to misclassification of HIV-2- infected individuals since HIV-1 and HIV-2 Western blots lack specificity for type-specific diagnosis because there is significant cross-reactivity to gag, pol, and env oligomeric proteins. PCR has been used as gold standard for HIV-2 diagnosis but it still remains a research tool and a commercial assay is not yet available [3,11,12].

The diagnosis of HIV-2 has important implications for the choice of antiretroviral treatment (ART) regimens, as HIV-2 strains are naturally resistant to non-nucleoside reverse transcriptase and fusion inhibitors and are, at least *in vitro*, less sensitive to some protease inhibitors [13]. Consequently, accurate diagnosis of HIV-2 infection is critical for clinical management of patients [14,15].

Mozambique is situated in southeastern Africa and is bordered by six countries: Tanzania, Malawi, Zambia, Zimbabwe, Swaziland and South Africa and gained independence from Portugal in 1975, after nearly five centuries of Portuguese occupation. Furthermore, in the last two decades, there have been a considerable number of people from West Africa, Europe, and Asia visiting and living in Mozambique. These migratory movements may have facilitated the introduction and spreading of HIV-2 in Mozambique. Despite the existence of sporadic reports, the prevalence of HIV-2 in Mozambique is largely unknown [16]. In this work, we utilized a combination of serological and molecular techniques to estimate the prevalence of HIV-2 infection in Maputo City, Mozambique.

## Materials and methods

### Study design and subjects

This was a cross-sectional study that enrolled individuals with a recent HIV diagnosis attending the Centro de Saúde do Alto Maé outpatient clinic in Maputo City, Mozambique. A total of 1,200 consecutive individuals were recruited to participate at the time of routine CD4+ T-lymphocyte (CD4+) count. After written informed consent, two EDTA tubes were collected from each volunteer for CD4+ count and HIV-2 screening test, both performed at the Instituto Nacional de Saúde (INS), Mozambique. Confirmatory serological testing and HIV-2 molecular characterization was conducted at the Federal University of Rio de Janeiro, Brazil. The study protocol was approved in 2009 by the Mozambican National Health Bioethics Committee under Protocol number 129/CNBS.

### HIV serological assays

All individuals enrolled in this study had been previously diagnosed as HIV infected according to the Mozambican algorithm for HIV diagnosis which employs two sequential HIV-1/2 rapid tests: the Determine HIV 1/2 test (Abbott Laboratories, Tokyo, Japan), used for screening and UniGold HIV test (Trinity Biotech, Ireland), used to confirm initial reactivity on the Determine assay. HIV infection is ascertained only if both assays are reactive. Of note, both assays have the HIV-2 gp36 antigen on the solid phase, and are able to detect HIV-2 specific antibodies.

In addition to the HIV-1/HIV-2 specific peptide-EIA (pepEIA) assay described below, two confirmatory serological assays were used in this study: INNO-LIA HIV I/II Score (Innogenetics, Gent, Belgium) and Bio-Rad New Lav Blot II (Bio-Rad, Marnes-la-Conquette, France).

In order to initially differentiate the reactivity against HIV-1 and HIV-2 we used the same assay described by Qiu *et all *[17]. Briefly, synthetic peptides, carrying 14 amino acid residues from the immunodominant regions of transmembrane proteins of HIV-1 (gp41) or HIV-2 (gp36) were synthesized with the addition of 7 non-specific residues at the amino-terminus (RGDKDGG) to increase the solubility of both peptides. HIV-1 or HIV-2 peptides were coated onto wells on separate microplates (Nalge Nunc International, Rochester, NY) at 5 μg/mL in 100 μL of 10mM phosphate buffer saline (PBS), pH 7.4 for 2 hr at 37°C. Following five washes with PBS, wells were blocked with 100 μL of BSA buffer (PBS with 0.05% Tween-20 and 0.5%BSA). Serum or plasma samples (diluted 1:100 in BSA buffer) were added to the coated wells and incubated for 1 hr at 37°C. After the wash, bound antibodies were detected by goat anti-human IgG peroxidase conjugate (30 min at 37°C, diluted in BSA buffer), followed by substrate (TMB) incubation for 25 min at room temperature.

The cutoff values were arbitrarily set at 0.5 OD for both EIAs; all negative specimens were below the cutoff value. The criteria for interpreting results were as follows: 1) HIV-1 OD ≥ 0.5 and HIV-2 OD < 0.5 were considered HIV-1 reactive only; (2) HIV-2 OD ≥ 0.5 and HIV-1 OD < 0.5 were considered HIV-2 reactive only; 3) HIV-1 OD and HIV-2 OD ≥ 0.5 were considered HIV-1 and HIV-2 dually reactive and; 4) HIV-1 OD and HIV-2 OD < 0.5 were considered non reactive. Samples dually reactive were further diluted 1:10 (to a final serum dilution of 1:1,000) and retested on the pepEIA, and the results were interpreted as above.

### Molecular detection and characterization

Buffy coats from samples suspected of having HIV-2 were used for DNA extraction using a blood DNA extraction kit (QIAamp, Qiagen, Hilden, Germany), according to the manufacturer's instructions. Detection of HIV-2 proviral DNA was carried out using nested polymerase chain reaction (PCR) of region *pol (protease and transcriptase reverse) *with some modifications to the protocol described by Colson *et al *[14]. Nested PCR was performed using 5 μL of extracted genomic DNAThe PCR conditions were 2,5U of Taq Platinum DNA polymerase (Invitrogen Life Technologies, Carlsbad, Calif), 1× polymerase buffer, 0.4 mM of each deoxynucleoside triphosphate, 3mM of Mgcl_2_, and 25 pmol of the outer primers (forward: H2Mp1, 5'-GGGAAAGAAGCCCCGCAACTTC-3';reverse: H2Mp2, 5'-GGGATCCATGTCACTTGCCA-3') in the first round. This amplification was carried out in a 50 μl reaction mixture to obtain a genomic fragment of 1,753 bp. The cycling conditions were 94°C for 2 min, followed by 40 cycles of 94°C for 30sec, 56°C for 45 sec, and at 72°C for 2 min) with one final cycle of 72°C for 7 min. The 2nd round was performed using 5 μL of the previous amplification product and 25 pmol of the inner primers (forward: H2Mp3, 5'-ACTTACTGCACCTCGAGCA-3'; reverse: H2Mp4, 5'-CCCAAATGACTAGTGCTTCTT-3'). Five microliters of the amplicon of the first-round were added in the second-round of PCR and performed under the same conditions of the 1st PCR to obtain a genomic fragment of 1,507 bp. Amplicons were detected by electrophoresis on 1,5% agarose gel and visualized under UV light after ethidium bromide staining.

To detect HIV-1 nested PCR was designed to amplify a 1000-bp fragment encompassing 297 bp of the protease gene and 703 bp of the RT gene. The conditions were 2.5 mM MgCl_2_, 0.4 mM of each deoxynucleotide triphosphate, 25 pmol of each primer (forward: K1F, 5'-5' CAGAGCCAACAGCCCCACCA -3'; reverse: K2R, 5'- TTTTCCCACTAACTTCTGTCATTGAC-3'), and 1.25U of Taq Platinum DNA polymerase (Invitrogen, CA, USA), 1x polymerase buffer and 5 μL of DNA genomic extracted in a final volume of 50 μL. The thermal conditions of amplification in the thermocycler device (ABI 9700, Applied Biosystems, USA) were: 94°C for 3 min, 5 cycles of denaturation at 94°C for 1 min, annealing at 55°C for 45 sec. and extension at 72°C for 2 min followed by 30 cycles of denaturation at 94°C for 1 min, annealing at 57°C for 45 sec. and extension at 72°C for 2 min. and incubation at 72°C for 7 min. The secondround was done with 3 μL of the amplicon of the first-round and 25 pmol of inner primers (forward: DP10, 5'- 5'CAACTCCCTCTCAGAAGCAGGAGCCG 3'; reverse: RT12 5'- ATCAGGATGGAGTTCATAACCCATCCA-3') in the same conditions of the first-round. Amplicons of 1000bp were detected by electrophoresis on 1% agarose gel and visualized under UV light after ethidium bromide staining [17-19].

For each run, HIV-1 and HIV-2 positive samples, in addition to a genomic DNA from an individual seronegative for HIV-1/2, were used as controls. The gp41 immunodominant region (IDR) was sequenced following the protocol previously described [20].

For sequencing analysis, PCR products corresponding to 1000 bp and 1507 bp of HIV-1 and HIV-2 pol region respectively were purified using QIAamp PCR purification kit (Qiagen) according to the manufacturer's instructions. The amplicons were directly sequenced using the BigDye Terminator v3.1 Cycle Sequencing Kit, with a 3730 Automated DNA Sequencer (Applied Biosystems, USA).

Sequences were edited and aligned using BioEdit program v5.0.9. (Department of Microbiology, North Carolina State University, USA), and Clustal W algorithm, respectively. Neighbor-joining (NJ) tree was built on Mega 4.0 software. The NJ tree was evaluated by bootstrap analysis of 1,000 replicates. The mean genetic distance between the Mozambican HIV-2 samples was determined using substitution model on Mega 4.0 software.

### Statistical analysis

Confidence Interval (CI) was used to address precision of the proportion estimates and the degree of confidence was set to 90% [21].

## Results

**Figure 1:**
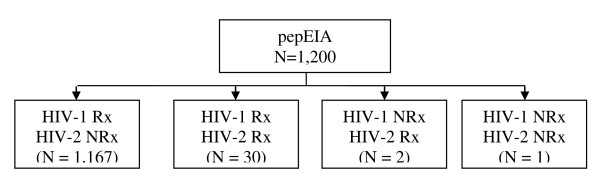
**Results of the pepEIA screening test for HIV types 1 and 2 in 1,200 HIV infected patients followed-up at the Alto Maé outpatient clinic**. Rx = pepEIA reactive and NRx = pepEIA non-reactive.

We initially screened 1,200 specimens by parallel testing with HIV-1 and HIV-2 pepEIA (Figure 1). One sample (FEH0045) was non-reactive to the gp41 and gp36 derived peptide. Further testing with the Vironostika HIV Uni-Form II plus O and the HIV-1 Western blot was also negative. Consistently with this serological finding, a PCR reaction set to amplify the reverse trasncriptase and protease regions from HIV-1 and HIV-2 was negative.. This sample was removed from analysis.

A total of 1,167 specimens were reactive only on the HIV-1 gp41 pepEIA after the initial screening. Thirty samples were simultaneously reactive to gp41 and gp36 peptides and after re-testing these specimens with a 1:10 dilution, 17 became reactive to HIV-1 peptide only. The remaining 13 samples remained reactive to both HIV-1 and HIV-2 peptides (Table 1). Finally, two samples were reactive with the gp36 peptide only after the initial screening. For these last two samples, HIV-2 reactivity persisted after 1:10 specimen dilution.

**Table 1:** Serological and genomic tests results of 32 patients reactive on the pepEIA HIV-2 screening assay

	**Result of pepEIA screening**^**a**^			Result of confirmatory assay							Result of nested PCR (Subtyping)	
						
				**INNO-LIA HIV 1/2**^**b**^								
**Patient** **code**				**HIV-1**			**HIV-2**		**HIV type**			
						
	**HIV-1 OD**	**HIV-2 OD**	**HIV-2 OD (1/10)**^**d**^	**p24**	**gp41**	**gp120**	**gp36**	**gp105**		**New** **LAV Blot**^**c **^**II (HIV-2)**	**HIV-1**	**HIV-2**

FEH0251	2578	563	155	3	3	3	neg	neg	1	nd	POS (C)	Neg

FEH0049	2886	592	356	3	3	3	neg	neg	1	nd	POS (C)	Neg

FEH0220	2815	624	196	3	3	3	neg	neg	1	nd	POS (C)	Neg

FEH0105	2820	694	283	3	3	3	neg	neg	1	nd	POS (C)	Neg

FEH0345	2804	713	230	3	3	3	neg	neg	1	nd	POS (C)	Neg

FEH0047	3024	718	310	3	3	3	2	neg	1-2	nd	POS (C)	Neg

FEH0190	2972	937	144	3	3	3	1	neg	1-2	nd	POS (C)	Neg

FEH0027	2979	947	273	2	3	3	neg	neg	1	nd	POS (C)	Neg

FEH0596	2892	962	160	3	3	3	neg	1	1-2	Indeterm	POS (C)	Neg

FEH0281	2539	1086	296	3	3	3	2	neg	1-2	POS	POS (C)	Neg

FEH0226	3008	1275	416	3	3	2	2	neg	1-2	POS	POS (C)	Neg

FEH0210	2820	1619	393	3	3	3	1	neg	1-2	POS	POS (C)	Neg

FEH0244	2805	1695	291	2	3	+/-	2	neg	1-2	Indeterm	POS (C)	Neg

FEH1044	2645	1764	446	3	3	3	neg	neg	1	nd	POS (C)	Neg

FEH0327	2885	2156	389	3	3	2	neg	neg	1	nd	POS (C)	Neg

FEH0700	2405	699	116	+/-	4	2	neg	2	1-2	nd	POS (C)	Neg

FEH0687	2799	1485	279	1	4	3	neg	neg	1	nd	POS (C)	Neg

FEH0086	2959	1426	755	3	3	3	neg	neg	1	nd	POS (C)	Neg

FEH0394	2794	1794	864	3	3	3	1	neg	1-2	POS	POS (C)	Neg

FEH1049	3010	2146	729	3	3	3	neg	neg	1	nd	POS (C)	Neg

FEH0875	2859	2222	702	2	3	3	neg	neg	1	nd	POS (C)	Neg

FEH0982	2412	2261	1425	3	3	3	neg	neg	1	nd	POS (C)	Neg

FEH0404	3031	2262	1054	3	3	3	neg	neg	1	POS	POS (C)	Neg

FEH0857	2837	2272	1481	3	3	2	neg	neg	1	nd	POS (C)	Neg

FEH0646	3008	2364	573	3	3	3	neg	neg	1	POS	POS (C)	Neg

FEH1196	3017	2620	1145	2	3	3	neg	neg	1	Indeterm	POS (C)	Neg

FEH1097	2996	2662	547	3	3	3	neg	neg	1	nd	POS (C)	Neg

FEH1040	2996	2697	672	3	3	3	neg	neg	1	nd	POS (C)	Neg

FEH0681	2882	2069	2988	2	4	3	1	neg	1-2	nd	POS (C)	Neg

FEH0089	2858	2809	1600	3	3	3	3	3	1-2	POS	POS (C)	POS (A)

FEH0726	197	2051	922	neg	neg	neg	3	1	2	POS	Neg	POS (A)

FEH0924	194	2779	2080	+/-	neg	neg	2	3	2	POS	Neg	POS (A)

^a^Underlined numbers denotes reactivity on the pepEIA;

^b^Only reactivity to antigens of interest is reported;

^c ^nd, not tested; indeterm, indeterminate;

**^d ^**assay performed with sample further diluted 1/10

The 30 initially dually reactive samples and the two HIV-2 reactive samples were further tested on the INNO-LIA immunoblot assay (Table 1). All 30 pepEIA dually reactive samples reacted against all five HIV-1 specific proteins present on the INNO-LIA (gp120, gp41, p31, p24 and p17), except for sample FEH1196 that was negative for p17. The analysis of reactivity against HIV-2 proteins (gp105 and gp36) revealed that among the 17 samples that became HIV-2 negative after re-testing with 1:10 sample dilution in the pepEIA assay, gp36 alone was present on six samples, gp105 alone was present on two samples and both bands were absent for nine samples. Of the 13 samples that remained HIV-2 positive after pepEIA re-testing with 1:10 sample dilution, 10 did not react to gp36 or gp105 while two reacted against gp36 only. Solely one sample (FEH0089) reacted to both gp36 and gp105 HIV-2 proteins. The two samples reactive on gp36 pepEIA only, reacted to both HIV-2 gp36 and gp105 proteins. One of these specimens (FEH0726) did not react to any HIV-1 proteins present on the INNO-LIA assay while the other (FEH0924) showed weak reactivity to the HIV-1 p24 protein (Table 1).

**Figure 2:**
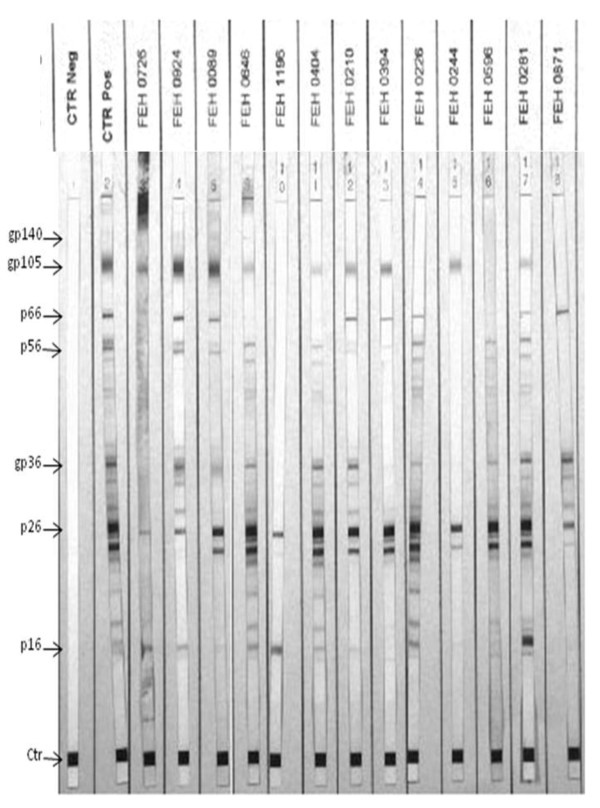
**Evaluation of HIV-2 Western blot profile (New Lav Blot II)**. The first two strips are negative (CTR Neg) and positive (CTR Pos) controls. The other lanes show samples from patients infected with HIV-2 (FEH 00726 and 0924), dually infected (FEH 0089) and dually reactive to HIV-1 and HIV-2 gp41/36 reactive, except for sample FEH0871 which is reactive to gp41 peptide only. The arrow indicates the molecular weights of viral proteins in kDa.

In order to explore the discriminatory power of HIV-2 Western blot we have analyzed the capacity of 9 HIV-1/HIV-2 dually reactive samples in recognizing HIV-2 proteins (Figure 2). The data indicated no discriminatory pattern and, on the basis of the manufacturer's WB interpretation criteria, all samples could be considered as HIV-2 positive. Likewise, the reactive on the HIV-1 Western blot from the 2 solely HIV-2 pepEIA reactive samples were evaluated. Sample FEH0726 did not react to any HIV-1 proteins; however, sample FEH0924 showed a strong reactivity to gp120 and weaker reactivity to p24, p31, p51 and p66 (data not shown).

All 32 samples showing reactivity to the HIV-2 gp36 peptide on the pepEIA were submitted to HIV-2 specific PCR. Only three samples were positive by PCR: one double- reactive on the pepEIA (FEH0089) and the two samples that reacted with the gp36 peptide only (FEH0726 and FEH0924). With the exception of these last two samples, all other 30 samples were also amplified using HIV-1 specific primers and the majority of those analyzed grouped with subtype C based on Stanford HIV Database subtyping tool (Table 1). Interestingly, the three HIV-2 PCR positive samples were the only samples reactive to both gp36 and gp105 on the INNO-LIA immunoblot. All HIV-2 positive samples (FEH0089, FEH0726 and FEH0924) *pol *gene (1507 bp fragment) were analyzed and sequences clustered with HIV-2 subtype A (Table 1) sequences after phylogenetic analysis.

In order to explore the cross-reactivity between HIV-1 and HIV-2 antibodies, a sub-group of HIV-1 positive samples (n = 22), some displaying cross-reactivity to HIV-2 (n = 16), were sequenced at the gp41 gene which included the 14 amino acid present at the gp41 immunodominant peptide used in the pepEIA. The gp41/36 IDR aa alignment showed that consensus sequence of 5 HIV-1 samples displaying no cross-reactivity to HIV-2 were not drastically different from 16 samples displaying cross-reactivity to HIV-2 IDR peptide. Of note, we found samples like FEH0871 and FEH0281 from both groups showing some similar aa signatures in IDR peptide to HIV-2 consensus such as A_27 _and R_29 _(Table 2).

**Table 2:** Analysis of amino acid sequence of the variable region of gp41 IDR peptide at positions 21 to 34 from 22 samples showing mono and dually reaction to HIV-1/HIV-2 gp41 IDR peptide

HIV-2 peptide		^21^L	N	S	W	G	C	A	F	R	Q	V	C	H	T^34^
		
HIV-1 peptide		L	G	I	W	G	C	S	G	K	L	I	C	T	T
FEH0856	non-xR	.	.	.	.	.	.	.	.	.	.	.	.	.	.
FEH0871	non-xR	.	.	M	.	.	.	.	.	**R**	.	.	.	.	.
FEH0882	non-xR	.	.	.	.	.	.	.	.	.	.	.	.	.	.
FEH1107	non-xR	.	.	M	.	.	.	.	.	.	.	.	.	.	.
FEH1117	non-xR	.	.	.	.	.	.	.	.	.	.	.	.	.	.

FEH0049	xR	.	.	L	.	.	.	.	.	.	.	.	.	.	.
FEH0105	xR	.	.	L	.	.	.	.	.	.	.	.	.	.	.
FEH0210	xR	.	.	.	.	.	.	.	N	.	.	.	.	.	.
FEH0327	xR	.	.	M	.	.	.	.	.	.	.	.	.	.	.
FEH0027	xR	.	.	.	.	.	.	.	.	.	.	.	.	.	.
FEH0047	xR	.	.	.	.	.	.	.	.	.	.	.	.	.	.
FEH0281	xR	.	.	V	.	.	.	**A**	.	.	H	.	.	.	.
FEH0596	xR	.	.	L	.	.	.	.	A	.	.	.	.	.	.
FEH0086	xR	.	.	.	.	.	.	.	.	.	.	.	.	.	.
FEH0394	xR	.	.	.	.	.	.	.	.	.	.	.	.	.	.
FEH0404	xR	.	.	M	.	.	.	.	.	.	.	.	.	.	.
FEH0646	xR	.	.	L	.	.	.	.	.	.	.	.	.	.	.
FEH0875	xR	.	.	.	.	.	.	.	.	.	.	.	.	.	.
FEH0982	xR	.	.	M	.	.	.	.	.	.	F	.	.	.	.
FEH1049	xR	.	.	.	.	.	.	.	.	.	.	.	.	.	.
FEH1097	xR	.	.	.	.	.	.	.	.	.	.	.	.	.	.
FEH0089	DI	.	.	.	.	.	.	.	.	.	.	.	.	.	.

non-xR - HIV-1 positive samples that did not show cross-reactivity to gp36 peptide on pepEIA; xR - HIV-1 positive samples that showed cross-reactivity to gp36 peptide on pepEIA; DI - HIV-1 and HIV-2 dually infected sample. Dots mean similarity between sequences and bold underlined letters correspond to positions with HIV-1 genetic divergence but similar amino acid to HIV-2.

## Discussion

This study found a low prevalence (0.25%, CI 90% 0.01-0.49) of HIV-2 in Maputo City, Southern Mozambique among HIV positive population attending ARV clinics. Additionally, of the three patients with HIV-2 infection, one was co-infected with HIV-1 subtype C. The number of dually infected individuals was lower than the HIV-2 infected subjects (one versus two). Barreto *et al *(1993) described the presence of individuals infected with HIV-2 in the city of Maputo since the late 80's using serological testing, and claimed that the entry of this virus was due to post war movement of population growth especially among countries of Portuguese language in this continent [22]. The overall prevalence of HIV-2 infected individuals found in our study is very low when compared to figures from West Africa where HIV-2 and dual infections account for 1 to 10%. This is similar to what is shown in West Africa where HIV-1 epidemic is increasing and overtaking HIV-2 infections [3,23-26].

Phylogenetically, all three HIV-2 specimens segregated as subtype A and due to a large (> 10%) genetic distance between them it is likely that these patients represent three different independent transmission events of HIV-2 in country. The presence of subtype A isolates in Mozambique fits with the hypothesis of introduction of HIV-2 from West Africa, where this variant dominates the epidemic. In fact, subtype A is the major HIV-2 variant representing the epidemic in Portuguese Speaking countries in West Africa such as Guine Bissau and Cape Verde [27-30,12].

On the other hand, EIA using IDR peptides were able to distinguish HIV-1 and HIV-2 infection with a sensitivity of 100% and specificity of 97.6% (90% CI 99.7% to 99.9%). There was a high proportion of cross-reactivity between HIV-1 and HIV-2 IDR peptides from sera of individuals infected with HIV-1. It seems that sera of HIV-2 mono-infected individuals solely recognize HIV-2 related IDR peptide in our pepEIA assay. The cross-reactivity of some HIV-1 infected individuals can be a source of false "dual infection" diagnosed in many publication based solely in IDR peptides EIA screening. In this sense, our data are in agreement with Ciccaglione *et al *[31]. This cross-reactivity can be minimized by 1:10 serum dilution in pepEIA which is an alternative strategy to identify false-reactive HIV-2 samples. Our data clearly demonstrated that the presence of gp105 and gp36 bands in INNO-LIA assay was strongly associated with HIV-2 infection. However, the same is not true when solely one of these bands is recognized. In our study the PCR reaction targeting HIV-2 pol region was very sensitive and specific to identify truly HIV-2 infected individuals. This fact led us to conclude that gp41/36 IDR pepEIA can be a good screening method followed by INNO-LIA and PCR as confirmatory tests for HIV-2 infection. This algorithm of test seems to be very robust and enable us to identify 3 HIV-2 infections among 1199 HIV positive sera.

The prevalence of HIV-2 in Mozambique and the boundary countries need to be monitored throughout the time to have a better understanding of its trend and geographical distribution. Limited information exists regarding commercial serological assays for HIV-2 diagnosis [32,33]. Therefore, the continuous survey for HIV-2 need to be incorporated at serological and molecular level in HIV surveillance programs in sub-Saharan countries. Guidelines for ART and algorithms for HIV diagnosis must take into account the prevalence of HIV-2 infection in a country. The new testing algorithm proposed here can be implemented in regions where HIV-2 is reported to circulate in order to optimize the 1^st ^line ARV regimens as well PMTCT interventions.

## Competing interests

The authors declare that they have no competing interests.

## Authors' contributions

CA, and RC carried out the molecular genetic studies, AT participated in the sequence alignment and drafted the manuscript. CM, and DC carried out the immunoassays. NB, and CW participated in clinical sites selecting patients. OCFJr participated in the design of the study and performed the statistical analysis. IJ and At FG conceived of the study, and participated in its design and coordination. All authors read and approved the final manuscript
